# The Interaction of Deworming, Improved Sanitation, and Household Flooring with Soil-Transmitted Helminth Infection in Rural Bangladesh

**DOI:** 10.1371/journal.pntd.0004256

**Published:** 2015-12-01

**Authors:** Jade Benjamin-Chung, Arifa Nazneen, Amal K. Halder, Rashidul Haque, Abdullah Siddique, Muhammed Salah Uddin, Kim Koporc, Benjamin F. Arnold, Alan E. Hubbard, Leanne Unicomb, Stephen P. Luby, David G. Addiss, John M. Colford

**Affiliations:** 1 School of Public Health, University of California, Berkeley, Berkeley, California, United States of America; 2 Centre for Communicable Diseases, International Centre for Diarrhoeal Disease Research, Bangladesh, Dhaka, Bangladesh; 3 Children Without Worms, Task Force for Global Health, Atlanta, Georgia, United States of America; 4 Department of Medicine, Stanford University, Stanford, California, United States of America; National Institute of Parasitic Diseases, CHINA

## Abstract

**Background:**

The combination of deworming and improved sanitation or hygiene may result in greater reductions in soil-transmitted helminth (STH) infection than any single intervention on its own. We measured STH prevalence in rural Bangladesh and assessed potential interactions among deworming, hygienic latrines, and household finished floors.

**Methodology:**

We conducted a cross-sectional survey (n = 1,630) in 100 villages in rural Bangladesh to measure three exposures: self-reported deworming consumption in the past 6 months, access to a hygienic latrine, and household flooring material. We collected stool samples from children 1–4 years, 5–12 years, and women 15–49 years. We performed mini-FLOTAC on preserved stool samples to detect *Ascaris lumbricoides*, *Enterobius vermicularis*, hookworm, and *Trichuris trichiura* ova. Approximately one-third (32%) of all individuals and 40% of school-aged children had an STH infection. Less than 2% of the sample had moderate/heavy intensity infections. Deworming was associated with lower *Ascaris* prevalence (adjusted prevalence ratio (PR) = 0.53; 95% CI 0.40, 0.71), but there was no significant association with hookworm (PR = 0.93, 95% CI 0.60, 1.44) or *Trichuris* (PR = 0.90, 95% CI 0.74, 1.08). PRs for hygienic latrine access were 0.91 (95% CI 0.67,1.24), 0.73 (95% CI 0.43,1.24), and 1.03 (95% CI 0.84,1.27) for *Ascaris*, hookworm, and *Trichuris*, respectively. Finished floors were associated with lower *Ascaris* prevalence (PR = 0.56, 95% CI 0.32, 0.97) but not associated with hookworm (PR = 0.48 95% CI 0.16,1.45) or *Trichuris* (PR = 0.98, 95% CI 0.72,1.33). Across helminths and combinations of exposures, adjusted prevalence ratios for joint exposures were consistently more protective than those for individual exposures.

**Conclusions:**

We found moderate STH prevalence in rural Bangladesh among children and women of childbearing age. This study is one of the first to examine independent and combined associations with deworming, sanitation, and hygiene. Our results suggest that coupling deworming with sanitation and flooring interventions may yield more sustained reductions in STH prevalence.

## Author Summary

Soil-transmitted helminth infections remain prevalent in many low-resource areas of the world. The World Health Organization recommends that schoolchildren in countries where these infections remain common receive deworming medication two times a year. However, previous research has shown that people who live in countries where these infections are common are frequently reinfected within 6 months of taking deworming medication. Programs that improve sanitation and hygiene might help complement deworming programs to reduce reinfection and prevent transmission. We conducted a survey of women and children in rural Bangladesh to understand potential sanitation and hygiene interventions that could complement deworming. We found that people who took deworming medication and had access to a hygienic latrine had a lower worm infection prevalence than people who only took deworming medication. We also found that people who took deworming medication and had a house with a finished floor had a lower prevalence than people who only took deworming medication. Our results suggest that coupling deworming with sanitation and flooring interventions may be a more successful strategy for reducing STH transmission in the long run.

## Introduction

The World Health Organization recommends mass drug administration of albendazole or mebendazole to school-aged children to control soil-transmitted helminths in endemic countries; in addition, they recommend improved sanitation and hygiene to ensure long-term sustainability of deworming efforts [1]. While anthelminthics are highly efficacious in the short term, it is estimated that within six months, on average, 68% of those treated become reinfected with *Ascaris*, 67% with *Trichuris*, and 55% with hookworm [2]. Improvements to sanitation [3–8] and installation of finished flooring in homes may contribute to more sustainable reductions in STH transmission and prevalence either when delivered alone or in combination with deworming. However, to date, STH control has largely been a separate enterprise from the control of enteric pathogens through sanitation and hygiene interventions [9]. There has been a call to consider the joint effects of anthelminthic treatment and water, sanitation, and hygiene interventions [7,9], yet few studies have done so rigorously [10–13]. Considering the increased concerns about the potential for resistance to anthelminthic drugs [14,15], it is increasingly important to identify interventions that may more sustainably reduce transmission and minimize the duration of mass drug administration campaigns. Evidence that the combination of deworming and improved household environmental conditions results in greater risk reductions than either alone (i.e., evidence of a synergistic interaction) would motivate the delivery of combined interventions to more sustainably reduce the incidence and transmission of STH. Furthermore, such evidence could inform the targeting of deworming interventions to households with environmental conditions in which deworming is most effective.

Several studies have suggested that improved sanitation can reduce the risk of STH infection by reducing the shedding of helminth ova into the environment, leading to reduced transmission [7,16–18]. Another potential intervention that may reduce transmission is the provision of finished floors (e.g., cement or wood floors). Since STH eggs must be deposited in the soil to reach their infective stages, providing finished floors to households to replace earthen floors may reduce transmission by reducing the number of infective stages in the living space. The few studies that have systematically explored whether finished flooring is associated with STH infection found evidence of reduced risk but did not adjust for potentially strong confounders, such as household wealth [19–22]. To date, no studies have formally explored interactions between water, sanitation, and flooring interventions and deworming [23].

In Bangladesh, the Ministry of Health and Family Welfare has implemented mass drug administration of mebendazole to control STH infection in schools in 27 out of 64 districts bi-annually since 2008. In addition, the Bangladesh Expanded Program on Immunization offers mebendazole to pre-school children in Bangladesh. To control lymphatic filariasis infection, the Ministry has offered albendazole and diethylcarbamazine in endemic areas each year to all individuals over 1 year of age who are not pregnant since 2001; as of 2008, the program has operated in 20 districts. Prior to mass drug administration, an estimated 80% of Bangladeshi school-age children in rural areas were infected with STH [24]. To our knowledge, there have not been any systematic surveys of STH prevalence since mass drug administration began in Bangladesh.

Our objectives were to estimate the prevalence of STH infection among children and women of childbearing age in rural Bangladesh and to estimate the separate and combined associations of deworming, hygienic latrines, and finished floors with STH prevalence.

## Methods

### Study population and sample

In this study, we collected stool samples from a subset of individuals who participated in an ongoing cross-sectional study led by the International Centre for Diarrhoeal Disease Research, Bangladesh (ICDDR,B) in rural Bangladesh and conducted a secondary analysis of survey data from that study. The original study evaluated the Sanitation Hygiene Education and Water Supply in Bangladesh (SHEWA-B) program, which was implemented by UNICEF and the Government of Bangladesh from 2007–2012. Local hygiene promoters visited mothers of children under five years old in underserved areas of rural Bangladesh and delivered key messages about safe water, sanitation, and hygiene practices. The program did not offer deworming or improvements to household flooring. In a small subset of villages with high poverty levels, the program offered subsidized latrines [25]. The data used in this cross-sectional study were collected in 2012 in 68 sub-districts, 19 districts, and 50 intervention and 50 control village clusters in rural Bangladesh to evaluate SHEWA-B in 2012. The intervention, selection of control areas, eligibility, and sampling of clusters have been described elsewhere [26]. In this study, we did not stratify by whether a respondent participated in SHEWA-B or lived in a control cluster because the impact evaluation of SHEWA-B found that there was no increase in access to improved latrines, safe disposal of feces, availability of a handwashing station, or safe drinking water storage among SHEWA-B participants [25]. Thus, we considered it unlikely that SHEWA-B participation would be a potential confounder of the association between our exposures of interest and STH infection. The field team collected stool samples and questionnaires about socio-demographic information and anthelminthic treatment in October 2012. In December 2012, they administered a questionnaire to the same households to measure access to hygienic latrines, finished floors, and other environmental exposures. Latrine and flooring status were ascertained following stool sample collection due to field logistics constraints and the need to complete stool collection prior to national mass drug administration in early November 2012.

### Stool specimen collection and analysis

In each cluster, stool was collected from individuals in 17 randomly selected households; this number was determined by the sample size calculations from the original SHEWA-B evaluation. Field workers aimed to stratify sample collection by age such that six people within each of the following age and sex categories provided stool in each cluster: children 1–4 years, children 5–14 years, and women 15–49 years. We stratified by these groupings because in Bangladesh pre-school children (1–4 years) are offered deworming through the Expanded Program on Immunization, and school-aged children (5–14 years) are offered deworming through a separate program administered by the Ministry of Health. We collected stool from women of childbearing age because our exposure assessment focused on household-level exposures, and in rural Bangladesh, women in this age group spend most of their time at home, so these exposures are more relevant for women than men. In addition, women of childbearing age are also more likely to be exposed to STH ova shed by young children than older women.

Field workers provided households with plastic sheets and stool collection tubes and returned within 24 hours to collect samples. They stored 1g of stool in 20 ml of 4% sodium acetate-acetic acid-formalin. The maximum time between defecation and stool processing was 12 hours. Samples were transported to Dhaka, Bangladesh, for laboratory analysis at the International Centre for Diarrhoeal Disease Research, Bangladesh.

Helminth ova were detected using mini-FLOTAC, a copromicroscopic diagnostic technique appropriate for preserved stool [27,28]. Laboratory staff centrifuged samples at 1500 RPM for 3 minutes and then discarded the supernatant and suspended the sedimented stool in 20 ml of flotation solution 2 (saturated sodium chloride). They then mixed the contents thoroughly and filled each of the two chambers of the mini-FLOTAC device with 1 ml of the mixed sample. Staff recorded the number of eggs of *Ascaris lumbricoides*, hookworm, *Trichuris trichiura*, and *Enterobius vermicularis* in each chamber. For each helminth, we averaged the number of eggs in each chamber and multiplied the number by a factor of 10 to quantify the number of eggs per gram of stool. Laboratory analyses were conducted within 9 months of stool sample collection.

### Outcome and exposure definitions

Outcomes included the presence of any helminth ova as well as the intensity of helminth infection. Moderate/high intensity infections were defined as ≥5,000 eggs/gram for *Ascaris*, ≥1,000 eggs/gram for *Trichuris*, and ≥2,000 eggs/gram for hookworm [29]. Exposures included access to a hygienic latrine, household flooring material (earth/bamboo or cement/wood), and self-reported deworming in the last six months. We defined hygienic latrines as flush latrines connected to a piped sewer system, septic tank, off-set pit latrine, pit latrine with slab and functional water seal, pit latrine with slab, lid and no water seal, or a composting latrine. We defined “unhygienic” latrines as those that would likely fail to separate feces from the environment effectively including flush latrines connected to canal or ditch, pit latrines with or without a slab, no or broken water seal or a hanging latrine. This definition was developed by the International Centre for Diarrhoeal Disease Research, Bangladesh (ICDDR,B) and is intended to more accurately categorize latrines that isolate feces from the environment in the Bangladeshi context than the commonly used World Health Organization Joint Monitoring Programme (JMP) definition [30] (see S1 Table). Respondents reported whether each person who provided a stool sample took deworming medication in the last six months. If so, they were asked approximately how many weeks or months ago they took deworming. Field workers recorded whether deworming was received as part of a campaign and the source of deworming (e.g. clinic, school).

We calculated the cluster-level deworming coverage as the percentage of respondents in the sample who reported taking deworming in the prior six months in a given cluster. To estimate cluster-level sanitation and finished floor coverage, we calculated the percentage of respondents with a hygienic latrine, finished household floor, or who reported deworming consumption in a cluster.

We controlled for the following potential confounders in statistical models: age, sex, household wealth, cluster-level wealth, mother's education level, and district of residence. We used information about household assets (e.g. refrigerator, mobile phone) to develop an index of household wealth using principal components analysis [31] (see S2 Table). Households in the lowest three quintiles of the first principal component were classified as lower household wealth and those in the highest two quintiles were classified as higher household wealth. Cluster-level wealth was calculated as the percentage of households in the two highest quintiles of household wealth.

### Sample size

Since age- and sex-specific STH prevalence estimates were not available for the study areas, we assumed the prevalence of all helminths to be 50%. We assumed a design effect of 2.6 based on intraclass correlation coefficients estimated in a study measuring STH infection in a similar population in India [32] since such information for Bangladesh was not available. Because our study was nested within the ongoing SHEWA-B evaluation, our calculations assumed a fixed sample size of 1,700 (100 village clusters x 17 individuals per cluster). Under these assumptions, the precision associated with an estimate of prevalence of 50% is ±4%.

### Statistical analysis

We calculated pooled and age- and sex-specific prevalence by species of helminth. To examine the association between prevalence and cluster-level variables, we produced scatter plots of the observed variables and used locally weighted scatter plot smoothing (LOWESS) with normal-based pointwise 95% confidence bands to explore patterns in each scatter plot [33]. We also estimated the intraclass correlation coefficient for each STH infection within each cluster using a one-way analysis of variance.

To estimate adjusted prevalence ratios we used modified Poisson regression [34] adjusting for the potential confounders defined above. For each of the three exposures, we adjusted for the other two exposures in each model (e.g., the models for deworming were adjusted for hygienic latrines and finished floors). We estimated robust standard errors clustered at the village level to account for potential within-village outcome correlation. We excluded individuals with missing outcomes from the analysis, which assumes that they were missing at random conditional on covariates in our model.

Standard statistical models for binary outcomes predict outcomes and assess statistical interaction on the multiplicative scale. In this study, we chose to estimate interaction on the additive scale, which is useful when one is interested in the extent to which a primary exposure may yield greater health improvements by introducing a secondary exposure. In our study population, we consider deworming to be a primary exposure due to ongoing school-based deworming activities in Bangladesh. Our approach allows us to assess whether secondary exposures in addition to deworming, such as access to hygienic latrines, were associated with lower STH prevalence than deworming alone [35]. Specifically, we estimated the relative excess risk due to interaction (RERI), a measure of additive interaction [36]. When the exposures of interest are associated with only a lower or higher prevalence, an RERI>0 indicates a synergistic interaction between exposures [37,38]. If exposures could be associated with either increased or decreased prevalence, then the RERI must be greater than 1 for synergistic interaction to be present [37,38]. Since we expected associations to be protective, we recoded variables prior to RERI calculation so that the stratum with the prevalence ratio furthest from the null was reassigned as the reference group [39]. Because data were clustered at the village level, we used the bootstrap and resampled clusters to estimate 95% confidence intervals [40,41]. We did not estimate confidence intervals for any point estimates for which there were strata with fewer than 5 units. Analyses were conducted in Stata version 12 and in R version 3.1.3.

### Ethics statement

This study was approved by the Institutional Review Board (IRB) at the Centers for Disease Control and Prevention (CDC) (Protocol #6061). Study participants provided written consent. The CDC IRB approved this consent procedure. We offered a single dose of mebendazole to all participants who provided a stool sample.

## Results

**Fig 1:**
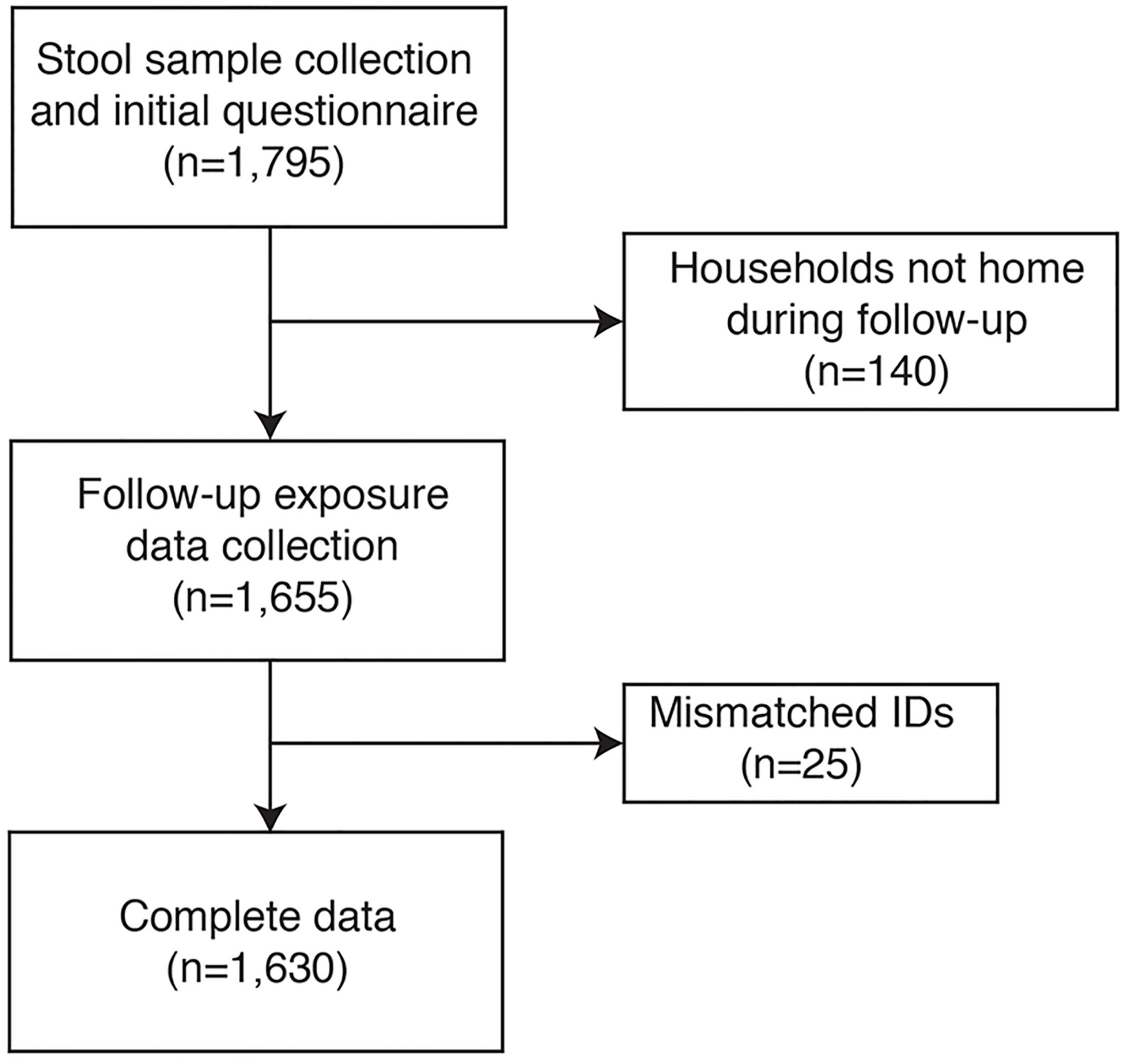
Data collected. This figure shows the number of surveys returned, samples analyzed, and final sample size for this analysis.

The field team collected stool samples and demographic information from 1,795 individuals in October 2012 and collected exposure information from 1,655 individuals in December 2012 (Fig 1). There were 1,630 individuals in the complete dataset; 140 individuals were not home during follow-up, and 25 identification numbers were mismatched between survey rounds. The number of missing observations for each variable of interest is listed in S3 Table.

Less than half (40%, n = 656) of mothers of the youngest child in each household had received at least a primary education. About a third of households (32%, n = 527) had access to a hygienic latrine, and 13% (n = 207) of households had finished floors. Respondents reported that 49% of children 1–4 years, 52% of children 5–14 years, and 21% of women of childbearing age took deworming medication in the prior six months. Child caregivers reported that less than half (47%) of school-age children took deworming drugs at school.

**Fig 2:**
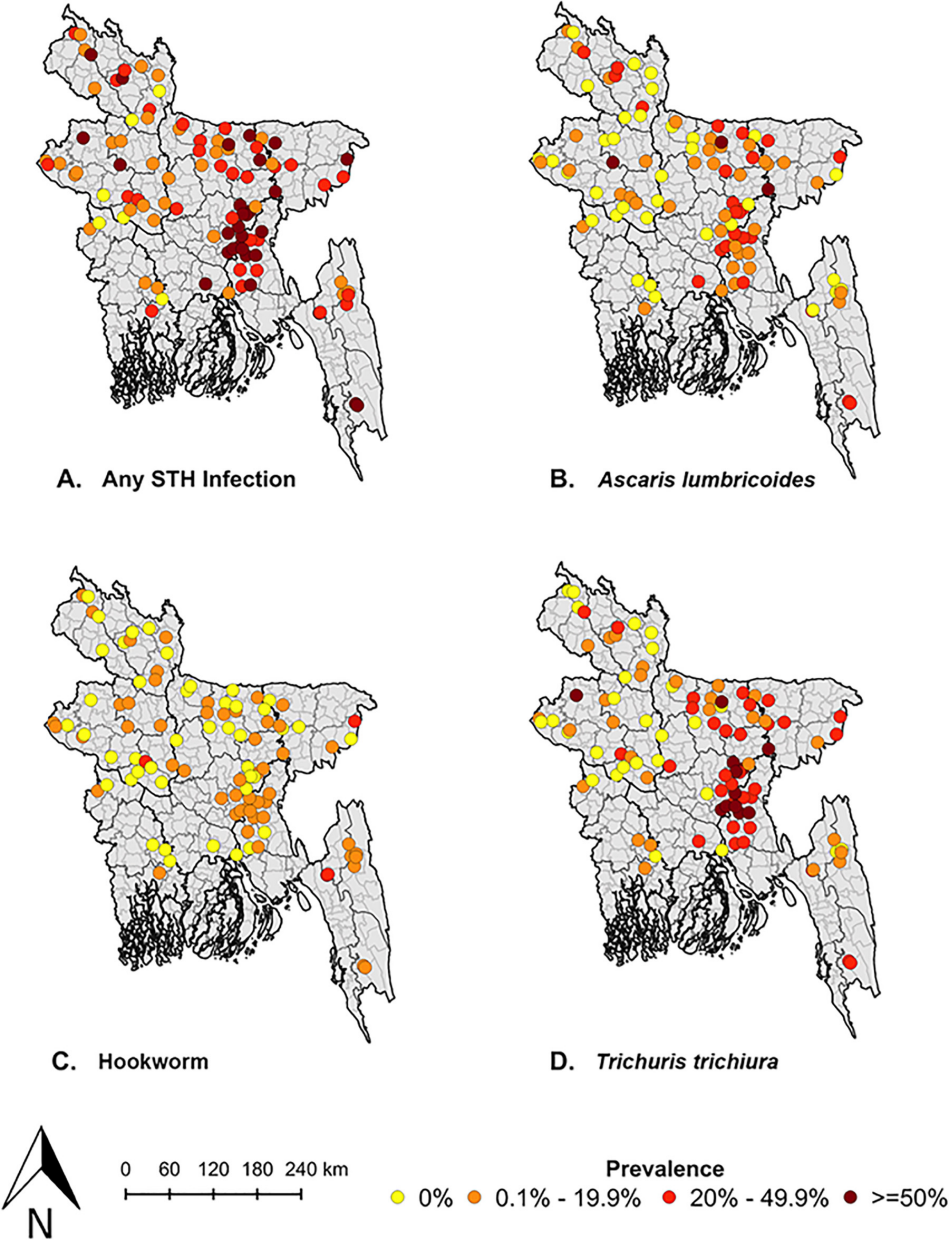
Map of soil-transmitted helminth prevalence in Bangladesh. We mapped STH prevalence in each study cluster for which valid GPS coordinates were available (n = 99). Panel A shows the cluster-level prevalence of any STH infection, Panel B shows the prevalence of *Ascaris lumbricoides*, Panel C shows the prevalence of hookworm, and Panel D shows the prevalence of *Trichuris trichiura*.

Approximately one-third (32%) of individuals sampled had an STH infection, and 9% had multiple infections (Table 1). Across all age groups, *Trichuris* was most prevalent, infecting 17% of children 1–4 years, 28% of children 5–14 years, and 18% of women of childbearing age. For all helminths and age groups, fewer than 2% had moderate/heavy intensity *Ascaris* or *Trichuris* infections; there were no moderate/heavy intensity hookworm infections. Prevalence of *Ascaris* and *Trichuris* were highest in areas in Dhaka and northern Chittagong divisions (Fig 2).

**Table 1:** Helminth infection prevalence by age, helminth, and infection intensity.

		Child 1–4 years (n = 549)	Child 5–14 years (n = 549)	Women 15–49 years (n = 532)	All ages (n = 1,630)
Female (%)		47.7	50.3	100	65.6
Dewormed in last six months (%)		49.3	52.4	20.9	41.0
Mean months since deworming		2.8	3.2	2.3	2.8
Source of deworming					
	Home/village	68.7	37.4	76.6	56.5
	Health clinic	26.9	15.7	22.5	21.4
	School	3.4	46.9	0.0	21.5
	Other	1.1	0.0	0.9	0.6
Any infection^a^		25.7	40.1	30.3	32.0
Multiple infections^a^		7.8	12.4	7.5	9.3
*Ascaris*					
	Prevalence (%)	12.9	14.4	11.8	13.1
	Mean eggs per gram	318	279	387	287
	No infection (%)	87.1	85.6	88.2	86.9
	Light infection (%)	11.8	12.6	10.7	11.7
	Moderate/heavy infection (%)	1.1	1.8	1.1	1.3
Hookworm					
	Prevalence (%)	2.6	7.7	6.4	5.5
	Mean eggs per gram	8	2	12	10
	No infection (%)	97.4	92.3	93.6	94.5
	Light infection (%)	2.6	7.7	6.4	5.5
	Moderate/heavy infection (%)	0.0	0.0	0.0	0.0
*Trichuris*					
	Prevalence (%)	17.1	27.5	18.2	21
	Mean eggs per gram	43	32	74	22
	No infection (%)	82.9	72.5	81.8	79
	Light infection (%)	16.8	26.4	18	20.4
	Moderate/heavy infection (%)	0.4	1.1	0.2	0.6
*Enterobius*					
	Prevalence (%)	0.01	0.05	0.02	0.03
	Mean eggs per gram	4	0	10	2
	No infection (%)	—	—	—	—
	Light infection (%)	—	—	—	—
	Moderate/heavy infection (%)	—	—	—	—

aIncludes *Enterobius* infections

We estimated associations between individual exposures and STH infection (Table 2). Deworming was associated with 47% lower *Ascaris* prevalence (95% CI 29%, 60%); adjusted associations with hookworm and *Trichuris* were null. There were no statistically significant associations with hygienic latrine access for any helminth. Finished floors were associated with 44% lower *Ascaris* prevalence (95% CI 3%, 68%). There was no statistically significant association between finished floors and hookworm or *Trichuris*. Participation in SHEWA-B was not associated with STH infection (S4 Table).

**Table 2:** Prevalence ratios for deworming, hygienic latrine access, and finished floors.

		*Ascaris*	Hookworm	*Trichuris*
Exposure (yes vs. no)	n^a^	PR (95% CI)	PR (95% CI)	PR (95% CI)
Unadjusted				
Deworming	1622	0.60 (0.46,0.80)	0.79 (0.52,1.21)	1.02 (0.84,1.24)
Access to hygienic latrine	1629	0.78 (0.59,1.04)	0.60 (0.37,0.97)	0.93 (0.75,1.14)
Finished floor	1630	0.45 (0.26,0.77)	0.32 (0.12,0.86)	0.88 (0.66,1.19)
Adjusted^b^				
Deworming	1573	0.53 (0.40,0.71)	0.93 (0.60,1.44)	0.90 (0.74,1.08)
Access to hygienic latrine	1579	0.91 (0.67,1.24)	0.73 (0.43,1.24)	1.03 (0.84,1.27)
Finished floor	1580	0.56 (0.32,0.97)	0.48 (0.16,1.45)	0.98 (0.72,1.33)

^a^The number of observations was slightly lower for adjusted prevalence ratios because some observations of potential confounders had missing values.

^b^PRs estimated using modified Poisson regression and adjusted for age, sex, sub-district, household wealth, cluster-level wealth, and mother’s education level.

**Fig 3:**
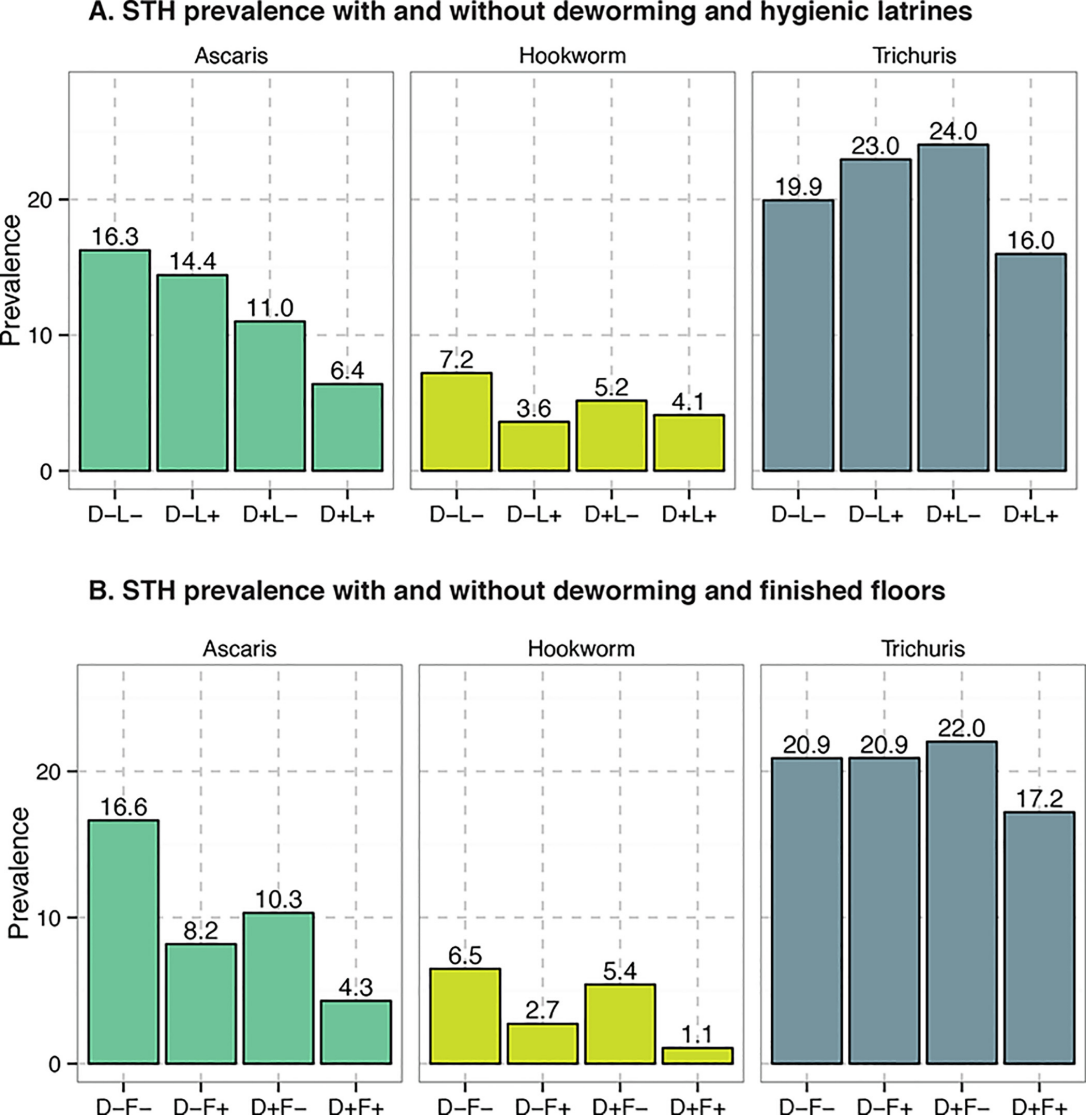
STH prevalence by exposure to deworming, hygienic latrines, and finished floors. Panel A shows the prevalence of each helminth among those who did not receive deworming (D-), those who did receive deworming (D+), those who did not have access to a hygienic latrine (L-), and those who did have access to a hygienic latrine (L+). Panel B shows the prevalence of each helminth among those who did not receive deworming (D-), those who did receive deworming (D+), those whose household did not have a finished floor (F-), and those whose household did have a finished floor (F+).

To explore potential interactions among deworming, hygienic latrines, and finished floors, we estimated adjusted prevalence ratios for each separately and in combination and the relative excess risk due to interaction RERI (Tables 3 and 4). The combination of deworming and hygienic latrines was associated with lower *Ascaris* and *Trichuris* prevalence than associations with separate exposures, although this association was only statistically significant for *Ascaris* (Table 3, Panel A; Fig 3). For example, deworming without hygienic latrine access (denoted D+L- in Fig 3) was associated with 45% lower *Ascaris* prevalence (95% CI 23%, 60%). *Ascaris* prevalence was equivalent for those with hygienic latrine access who did not take deworming (D-L+). The combination of deworming and hygienic latrine access (D+L+) was associated with 59% lower *Ascaris* prevalence (95% CI 27%, 76%). While individual and combined exposures were associated with lower hookworm prevalence, the associations did not follow the same pattern and were not statistically significant.

**Table 3:** Adjusted prevalence ratios for single and combined deworming and hygienic latrine access and relative excess risk due to interaction.

		*Ascaris*		Hookworm		*Trichuris*	
Deworming	Hygienic Latrine	n/N	aPR^a^ (95% CI)	n/N	aPR^a^ (95% CI)	n/N	aPR^a^ (95% CI)
-	-	106/652	(ref)	47/652	(ref)	130/652	(ref)
+	-	49/445	0.55 (0.40,0.77)	23/445	0.78 (0.48,1.28)	107/445	1.02 (0.81,1.27)
-	+	44/305	1.00 (0.72,1.39)	11/305	0.55 (0.28,1.08)	70/305	1.19 (0.92,1.54)
+	+	14/219	0.41 (0.24,0.73)	9/219	0.82 (0.38,1.75)	35/219	0.82 (0.59,1.14)
	RERI^b^		-0.36 (-2.18,0.58)		0.49 (-0.73,1.16)		-0.48 (-1.14,0.00)

^a^PRs adjusted for age, sex, household floor type, geographic district, household wealth, cluster-level wealth, and mother’s education level.

^b^Relative excess risk due to interaction (RERI). A RERI = 0 indicates no interaction on the additive scale, RERI>0 indicates synergistic interaction on the additive scale for monotonic aPRs, RERI>1 indicating synergistic interaction on the additive scale for non-monotonic aPRs, and RERI<0 indicates antagonistic interaction on the additive scale.

PRs adjusted for age, sex, hygienic latrine access, geographic district, household wealth, cluster-level wealth, and mother’s education level

95% Confidence intervals were not estimated because there were strata with fewer than 5 observations for this measure.

**Table 4:** Adjusted prevalence ratios for single and combined deworming and household finished floor and relative excess risk due to interaction.

		*Ascaris*		Hookworm		*Trichuris*	
Deworming	Finished floor	n/N	aPR^c^ (95% CI)	n/N	aPR^c^ (95% CI)	n/N	aPR^c^ (95% CI)
-	-	141/847	(ref)	55/847	(ref)	177/847	(ref)
+	-	59/572	0.53 (0.39,0.71)	31/572	0.93 (0.60,1.44)	126/572	0.91 (0.75,1.11)
-	+	9/110	0.60 (0.32,1.15)	3/110	0.54 (0.16,1.87)	23/110	1.12 (0.75,1.67)
+	+	4/93	0.28 (0.11,0.75)	1/93	0.27 (0.04,2.07)	16/93	0.85 (0.54,1.33)
	RERI^b^		0.56 (-3.64,2.40)		-1.14 (—,—)^d^		-0.18 (-1.48,0.37)

^a^PRs adjusted for age, sex, household floor type, geographic district, household wealth, cluster-level wealth, and mother’s education level.

^b^Relative excess risk due to interaction (RERI). A RERI = 0 indicates no interaction on the additive scale, RERI>0 indicates synergistic interaction on the additive scale for monotonic aPRs, RERI>1 indicating synergistic interaction on the additive scale for non-monotonic aPRs, and RERI<0 indicates antagonistic interaction on the additive scale.

^c^PRs adjusted for age, sex, hygienic latrine access, geographic district, household wealth, cluster-level wealth, and mother’s education level

^d^95% Confidence intervals were not estimated because there were strata with fewer than 5 observations for this measure.

Our estimates of the RERI for *Ascaris* and *Trichuris* were less than zero and not statistically significant, indicating no clear evidence of synergistic interaction between deworming and hygienic latrines on the additive scale. However, there was some suggestion of synergistic interaction for hookworm (RERI = 0.49; 95%CI -0.73, 1.16), although the confidence interval included 0.

For the combination of deworming and finished floors, we found the same pattern for all three helminths: the combination was associated with a lower prevalence than either exposure on its own (Table 3, Panel B; Fig 3). Deworming without finished floors (denoted D+F- in Fig 3) was associated with 47% lower *Ascaris* prevalence (95%CI 29%, 61%). Finished floors without deworming (D-F+) was associated with 40% lower *Ascaris* prevalence (95%CI -15%, 68%). The combination of deworming and finished floors (D+F+) was associated with 72% lower *Ascaris* prevalence (95%CI 89%, 25%). For *Ascaris*, the RERI was 0.56 (95%CI -3.64, 2.40). This pattern was also evident for hookworm and *Trichuris*.

**Fig 4:**
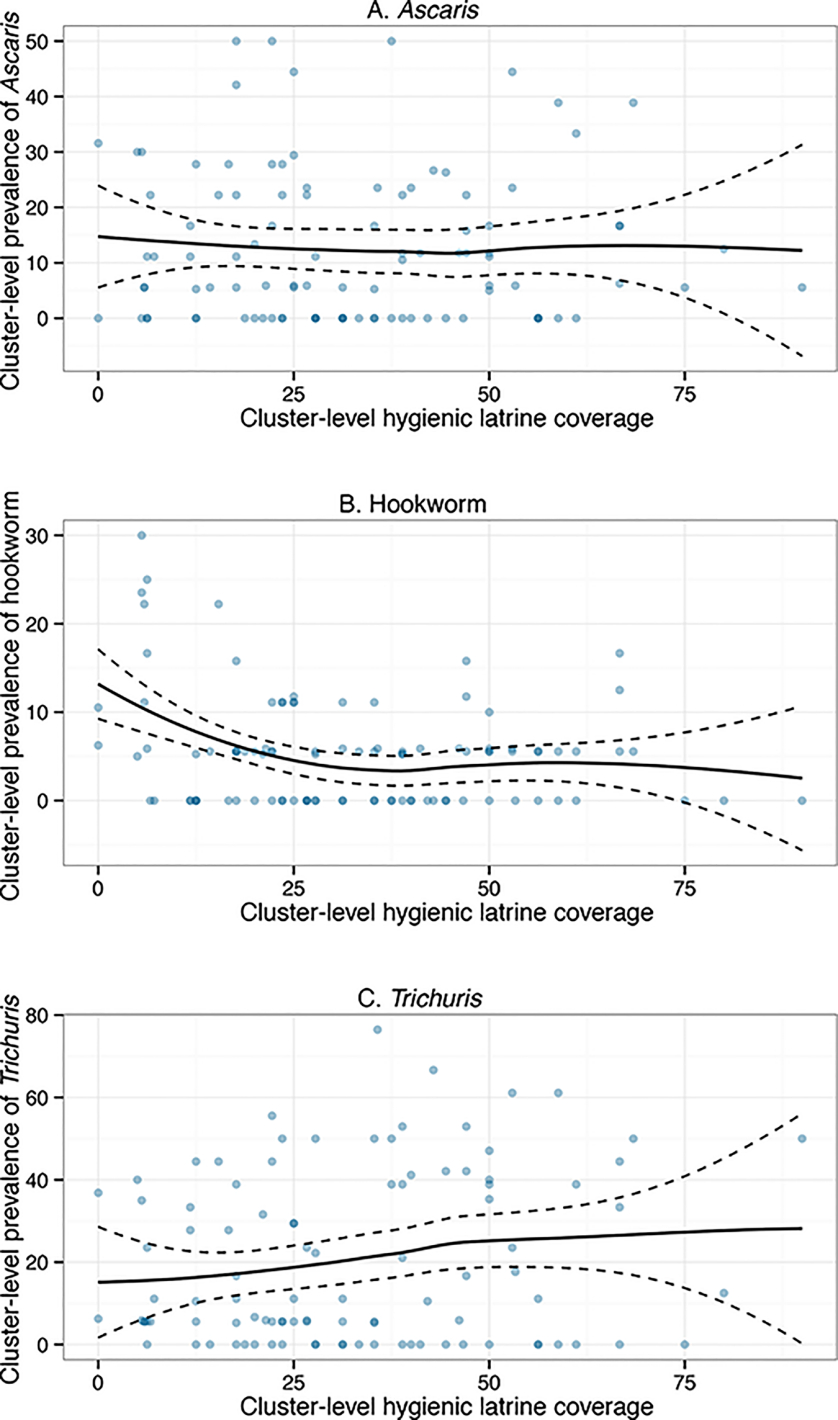
Cluster-level STH prevalence by cluster-level hygienic latrine coverage. Panel A shows the cluster-level prevalence of *Ascaris*, Panel B shows the prevalence of hookworm, and Panel C shows the prevalence of *Trichuris* by the proportion of respondents with hygienic latrines in each cluster.

We plotted the cluster-level prevalence of each helminth across the observed range of cluster-level deworming, hygienic latrine, and finished floor coverage (Figs 2 and S1 and S2). As hygienic latrine coverage increased, *Ascaris* and hookworm prevalence remained approximately the same and *Trichuris* prevalence increased slightly (Fig 4). As deworming and finished floor coverage increased, there was no substantial change in the prevalence of any helminth (S1 and S2 Figs). The village cluster level intraclass correlation coefficients were 0.11 (95% CI 0.08, 0.16) for *Ascaris*, 0.02 (95% CI 0.00, 0.05) for hookworm, and 0.21 (95% CI 0.15, 0.27) for *Trichuris*.

## Discussion

In this cross-sectional study of rural, low-income households in Bangladesh, where biannual national school-based deworming has been implemented for the last five years, the prevalence among school-aged children was 14% for *Ascaris*, 8% for hookworm, and 21% for *Trichuris*. Forty percent of school-aged children had at least one STH infection, and 12% had multiple infections. Approximately half the children under 15 years were reported to have taken deworming drugs in the prior six months. When we compared associations between STH infection and single versus combined exposures, we found that for deworming and hygienic latrines, *Ascaris* and *Trichuris* prevalence was lower among those with both exposures compared to those with only one exposure. Among those exposed to both deworming and finished floors, we found a lower prevalence for the combination of the two exposures than for either exposure separately. While our measures of additive interaction (the RERI) did not consistently indicate synergy and were not statistically significant, we consider the pattern of lower prevalence for combined exposures, particularly for deworming and finished floors, worthy of further exploration.

### Comparison of our prevalence estimates to prior estimates

The prevalence of any STH among school-aged children was 40% compared to 80% in rural areas reported by the Ministry of Health and Family Welfare prior to the initiation of school-based deworming [24]. The prevalence we observed is consistent with studies of school-based deworming with one-year follow-up and very high coverage [42–44]. Transmission theory [45–47] and empirical findings [48,49] suggest that prevalence decreases in pre-school age and adult populations when deworming coverage is high. We found that 26% of children 1–4 years and 30% of women of childbearing age had an STH infection. The similar prevalence in these two groups to the prevalence among school-aged children may suggest that, despite low levels of intensity, transmission is still ongoing in those population subgroups.

### Associations between STH and individual exposures

We observed associations between STH infection and deworming, finished floors, and hygienic latrines that were generally consistent with existing studies.

#### Deworming

We found that self-reported deworming was associated with a lower prevalence of *Ascaris* and hookworm, but the association was only significant for *Ascaris*. There was no association with *Trichuris* prevalence. These findings are consistent with those of randomized controlled trials estimating the efficacy of single-dose mebendazole and albendazole [50–52].

#### Hygienic latrines

Among those exposed to hygienic latrines, we found protective but not statistically significant associations with *Ascaris* and hookworm, but there was no association with *Trichuris*. We also examined associations between STH infection and access to any type of latrine and found similar results. These findings differ from those reported in two meta-analyses led by Ziegelbauer et al. and Strunz et al. examining sanitation and STH infection [7,53]. Ziegelbauer et al. identified 36 studies evaluating the association between latrine access and STH infection. They found protective associations for all three helminths for access to any type of latrine and for latrine usage [7]. The meta-analysis found evidence of publication bias, which is one possible explanation for our differing findings. Strunz et al. conducted a similar systematic review of water, sanitation, hygiene, and STH infection. They also found statistically significant protective associations between access to latrines of any kind and *Ascaris* and *Trichuris* but did not find an association with hookworm [53].

#### Finished floors

Among those exposed to finished floors, we observed a strong protective association with *Ascaris*, a protective association with hookworm that was not significant, and no association with *Trichuris*. The existing literature has for the most part reported that prevalence is lower for these helminths among those with finished floors; however, many previous studies did not adjust for household wealth, an important potential confounder of this association [19–22,54–56]. In this analysis, prevalence ratios for finished floors, adjusted for household wealth, were closer to the null than unadjusted estimates for each helminth, and for hookworm the association was no longer statistically significant following adjustment.

The null associations for all three individual exposures with *Trichuris*, the most prevalent helminth in this population, are noteworthy. There is some evidence that the prevalence of *Trichuris* decreases more slowly than that of other STH and that reinfection with *Trichuris* occurs more rapidly following intervention [57–59]. This may be because of longer survival of adult worms or because *Trichuris* has a higher reproductive rate than other STH [57,58]. The higher observed prevalence of *Trichuris* compared to *Ascaris* and hookworm in this study likely reflects these parasite-specific differences in biology and response to intervention.

### Associations between cluster-level exposures and STH prevalence

Contrary to what we would expect based on modeling studies [45–47], we did not find evidence of an association between STH prevalence and coverage of exposures at the cluster level. We expected that prevalence would decrease as the coverage of deworming, hygienic latrines, and finished floors increased. Such a pattern could be explained by herd effects of these exposures, which result from decreased shedding of infective stages in feces into the environment and reduced transmission [47]. Interestingly, our estimates of intraclass correlation coefficients suggest that cluster membership accounts for 25% of *Trichuris* prevalence and 11% of *Ascaris* prevalence; these findings are similar to the intraclass correlation coefficients reported in the literature [60]. Thus, village membership appears to be an important predictor of STH infection despite the lack of associations with cluster-level exposure coverage. It is possible that we did not find cluster-level associations because herd effects were small or absent for these exposures. It is also possible that such herd effects might have been higher if STH prevalence were higher. Alternatively, because we only collected data for 16 households per cluster on average, our sample might not have been sufficient to characterize cluster-level prevalence patterns. Additionally, variation in local population density in our sample could explain the lack of association because herd effects are likely to be stronger in densely populated areas. We did not account for population density in this analysis, and further work is needed to explore its role as a potential effect modifier.

### Limitations

This study is subject to several limitations. First, deworming consumption was self-reported over a six-month period. There is evidence that recall of medication consumption is under-reported with longer recall periods [61]. It is also possible that reporting was subject to courtesy bias so that deworming was over-reported. Given that it is unlikely that respondents knew if they had STH infection at the time of deworming reporting, we posit that it is unlikely that misclassification of deworming consumption differed by STH infection status. If non-differential misclassification occurred, it would bias point estimates towards the null [62]. It is also possible that individuals who were dewormed in the prior six months were reinfected prior to stool collection in this study. For this reason and because of the possible misclassification of deworming due to poor recall, the associations between deworming and STH infection do not necessarily measure the reduction in STH attributable directly to deworming.

Second, on average, stool samples were stored for 6 months prior to mini-FLOTAC analysis (min = 4, max = 8 months). This long storage period may have resulted in degradation of ova and underestimated prevalence, particularly for hookworm [63]. The sensitivity of mini-FLOTAC is estimated to be 75.5% for *Ascaris*, 79.2% for hookworm, and 76.2% for *Trichuris* [64]. Thus, given the sensitivity of the assay and the long storage period prior to analysis, our prevalence estimates are likely a lower bound of the true values. In addition, the sample size was powered to estimate prevalence but not to estimate interactions between exposures. Thus, our estimates of the RERI were in most cases underpowered, particularly for improved floors, which were relatively rare in this population.

Third, exposure measurement occurred following outcome measurement. This is suboptimal because it is possible that the outcome status of an individual would trigger a change in exposure status, leading to reverse causation. However, for the exposures measured—access to a hygienic latrine and finished floors—we consider it highly unlikely that the respondents' exposure status changed in the two months following outcome measurement. Furthermore, respondents were likely unaware of their outcome status throughout the study, so it is unlikely that they changed their sanitation infrastructure or flooring material because of their outcome status. Fourth, our analysis assumes that the sanitation and flooring infrastructure we observed were present within the past six months (the recall window for deworming consumption). We consider this to be a reasonable assumption; however, if these exposures were misclassified, prevalence ratios would be biased towards the null and the effects on measures of interaction would be unpredictable [65].

### Conclusions

This study provides the first estimates of STH prevalence following the initiation of mass drug administration for STH control among children and women of childbearing age in representative rural areas of Bangladesh. Our results suggest that combining deworming with sanitation and flooring interventions may yield greater reductions in STH prevalence than deworming alone.

## Supporting Information

S1 Checklist STROBE checklist. (PDF)

S1 Fig Cluster-level STH prevalence by cluster-level deworming floor coverage. Panel A shows the cluster-level prevalence of Ascaris, Panel B shows the prevalence of hookworm, and Panel C shows the prevalence of Trichuris by the proportion of respondents who took deworming in the past six months in each cluster.
(TIF)

S2 Fig Cluster-level STH prevalence by cluster-level finished floor coverage. Panel A shows the cluster-level prevalence of Ascaris, Panel B shows the prevalence of hookworm, and Panel C shows the prevalence of Trichuris by the proportion of respondents with finished floors in each cluster.
(TIF)

S1 Table Prevalence ratios for improved vs. hygienic latrine access. (DOCX)

S2 Table Means of each variable used in the principal components analysis by quintile of the index. (DOCX)

S3 Table Missing observations for outcome, exposure, and confounder variables. (DOCX)

S4 Table Association between SHEWA-B participation and STH infection. (DOCX)
