# Supplementary material for: The Interaction of Deworming, Improved Sanitation, and Household Flooring with Soil-Transmitted Helminth Infection in Rural Bangladesh
Source: PLoS Negl Trop Dis. 2015 Dec 1;9(12):e0004256. doi: 10.1371/journal.pntd.0004256 (PMC4666415; doi:10.1371/journal.pntd.0004256)
Supplement: S1 Checklist — (PDF) [file pntd.0004256.s001.pdf]

The interaction of deworming, improved sanitation, and household flooring with soil-transmitted helminth infection in rural Bangladesh

STROBE checklist for cross-sectional studies

	Item No	Recommendation	
**Title and abstract**	1	(*a*) Indicate the study's design with a commonly used term in the title or the abstract	✓
		(*b*) Provide in the abstract an informative and balanced summary of what was done and what was found	✓
**Introduction**			
Background/rationale	2	Explain the scientific background and rationale for the investigation being reported	✓ Lines 68-116
Objectives	3	State specific objectives, including any prespecified hypotheses	✓ Lines 118-121
**Methods**			
Study design	4	Present key elements of study design early in the paper	✓ Lines 126-172
Setting	5	Describe the setting, locations, and relevant dates, including periods of recruitment, exposure, follow-up, and data collection	✓ Lines 126-187
Participants	6	(*a*) *Cross-sectional study*—Give the eligibility criteria, and the sources and methods of selection of participants	✓ Lines 126-187
Variables	7	Clearly define all outcomes, exposures, predictors, potential confounders, and effect modifiers. Give diagnostic criteria, if applicable	✓ Lines 210-245
Data sources/ measurement	8*	For each variable of interest, give sources of data and details of methods of assessment (measurement). Describe comparability of assessment methods if there is more than one group	✓ Lines 125-194
Bias	9	Describe any efforts to address potential sources of bias	✓ Lines 237-245

Study size	10	Explain how the study size was arrived at	✓ Lines 249-258
Quantitative variables	11	Explain how quantitative variables were handled in the analyses. If applicable, describe which groupings were chosen and why	✓ Lines 262-275
Statistical methods	12	(*a*) Describe all statistical methods, including those used to control for confounding	✓ Lines 260-297
		(*b*) Describe any methods used to examine subgroups and interactions	✓ Lines 277-297
		(*c*) Explain how missing data were addressed	✓ Lines 274-275
		(*d*) *Cross-sectional study*—If applicable, describe analytical methods taking account of sampling strategy	✓ Lines 272-274
		(*e*) Describe any sensitivity analyses	✓ Tables S1, S4
**Results**			
Participants	13*	(a) Report numbers of individuals at each stage of study—eg numbers potentially eligible, examined for eligibility, confirmed eligible, included in the study, completing follow-up, and analysed	✓ Figure 1
		(b) Give reasons for non-participation at each stage	✓ Figure 1
		(c) Consider use of a flow diagram	✓ Figure 1
Descriptive data	14*	(a) Give characteristics of study participants (eg demographic, clinical, social) and information on exposures and potential confounders	✓ Table 1 and lines 319-325
		(b) Indicate number of participants with missing data for each variable of interest	✓ Table S3
Outcome data	15*	*Cross-sectional study*—Report numbers of outcome events or summary measures	✓ Table 1
Main results	16	(*a*) Give unadjusted estimates and, if applicable, confounder-adjusted estimates and their precision (eg, 95% confidence interval). Make clear which confounders were adjusted for and why they were included	✓ Table 2, Lines 237-245
		(*b*) Report category boundaries when continuous variables were categorized	✓ Lines 211-213

		(*c*) If relevant, consider translating estimates of relative risk into absolute risk for a meaningful time period	Not relevant
Other analyses	17	Report other analyses done—eg analyses of subgroups and interactions, and sensitivity analyses	✓ Line 225, 362
**Discussion**			
Key results	18	Summarise key results with reference to study objectives	✓ Line 440-464
Limitations	19	Discuss limitations of the study, taking into account sources of potential bias or imprecision. Discuss both direction and magnitude of any potential bias	✓ Line 556-594
Generalisability	21	Discuss the generalisability (external validity) of the study results	✓ Line 598-602
**Other information**			
Funding		Give the source of funding and the role of the funders for the present study and, if applicable, for the original study on which the present article is based	✓ Provided to PLOS NTDs editors
