# Supplementary figures and images for: The Interaction of Deworming, Improved Sanitation, and Household Flooring with Soil-Transmitted Helminth Infection in Rural Bangladesh
Source: PLoS Negl Trop Dis. 2015 Dec 1;9(12):e0004256. doi: 10.1371/journal.pntd.0004256 (PMC4666415; doi:10.1371/journal.pntd.0004256)

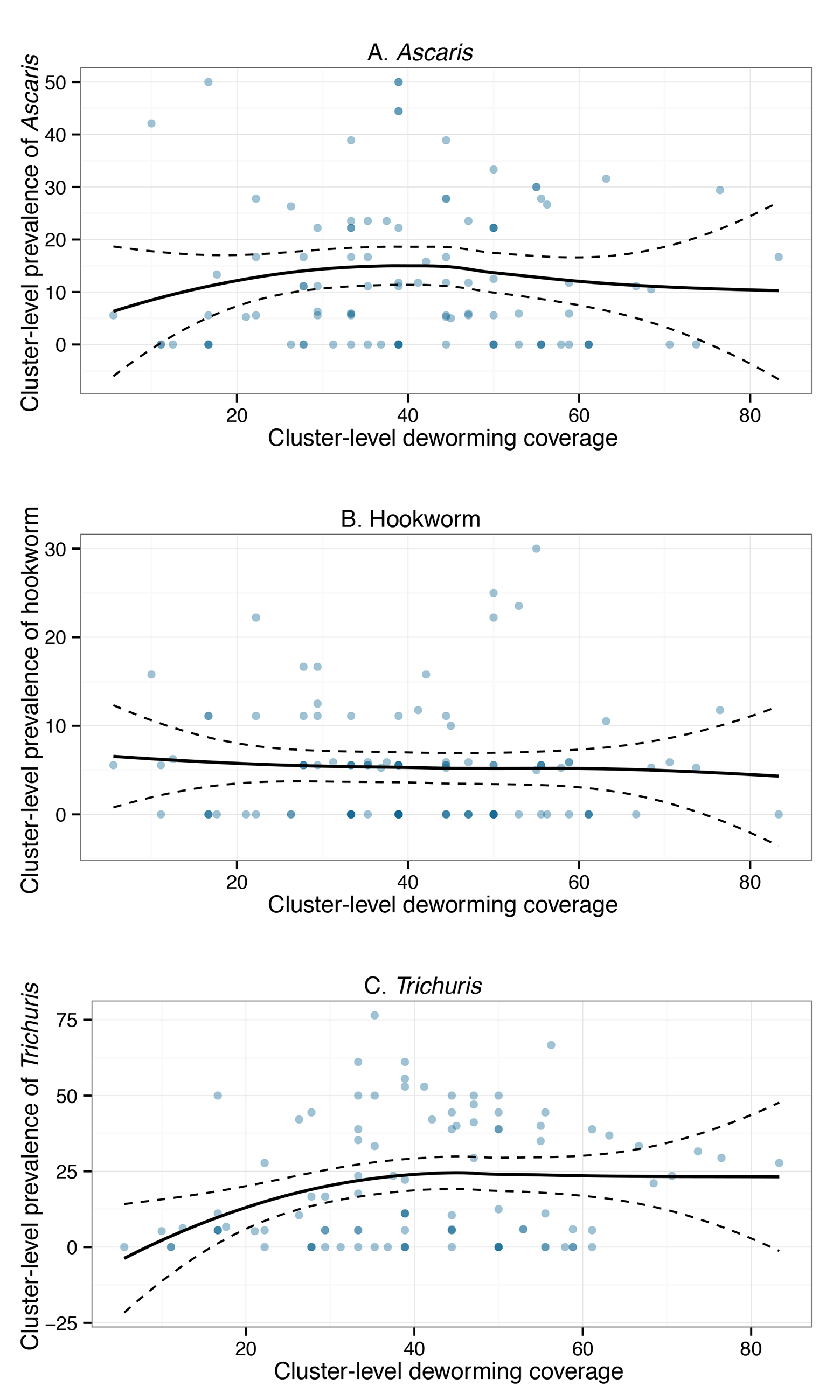

Supplement: S1 Fig — Panel A shows the cluster-level prevalence of Ascaris, Panel B shows the prevalence of hookworm, and Panel C shows the prevalence of Trichuris by the proportion of respondents who took deworming in the past six months in each cluster. (TIF) [file pntd.0004256.s002.tif]

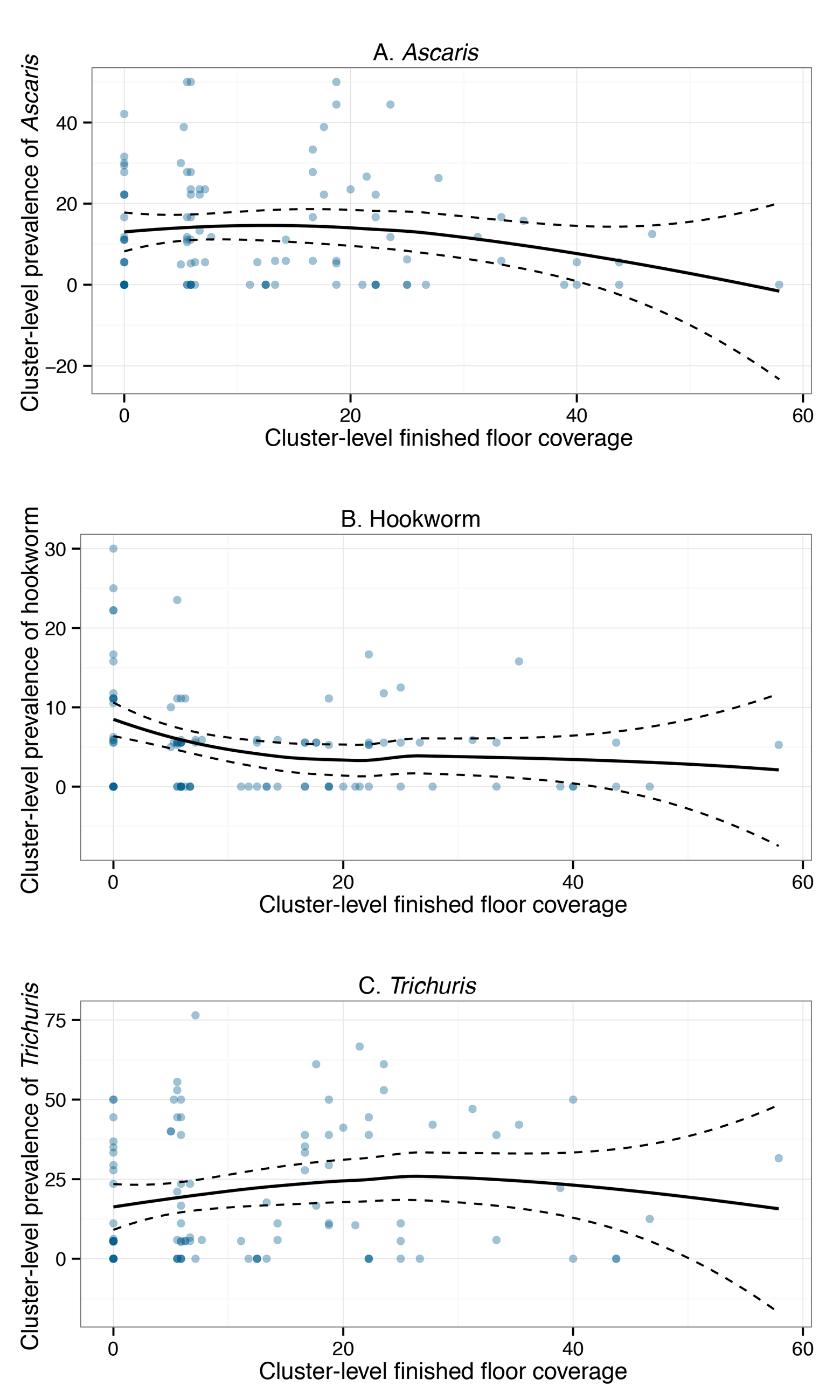

Supplement: S2 Fig — Panel A shows the cluster-level prevalence of Ascaris, Panel B shows the prevalence of hookworm, and Panel C shows the prevalence of Trichuris by the proportion of respondents with finished floors in each cluster. (TIF) [file pntd.0004256.s003.tif]
