# Supplementary material for: The Interaction of Deworming, Improved Sanitation, and Household Flooring with Soil-Transmitted Helminth Infection in Rural Bangladesh
Source: PLoS Negl Trop Dis. 2015 Dec 1;9(12):e0004256. doi: 10.1371/journal.pntd.0004256 (PMC4666415; doi:10.1371/journal.pntd.0004256)
Supplement: S1 Table — (DOCX) [file pntd.0004256.s004.docx]

**S1 Table. Prevalence ratios for improved vs. hygienic latrine access**

|  |  | *Ascaris* | Hookworm | *Trichuris* |
| --- | --- | --- | --- | --- |
|  | n | PR (95% CI) | PR (95% CI) | PR (95% CI) |
| Unadjusted |  |  |  |  |
| ICDDR,B-defined hygienic latrine ^a^ | 1629 | 0.78 (0.59,1.04) | 0.60 (0.37,0.97) | 0.93 (0.75,1.14) |
| JMP-defined improved latrine | 1629 | 0.99 (0.77,1.27) | 1.41 (0.93,2.12) | 1.04 (0.86,1.26) |
| Adjusted ^b^ |  |  |  |  |
| ICDDR,B hygienic latrine | 1579 | 0.91 (0.67,1.24) | 0.73 (0.43,1.24) | 1.03 (0.84,1.27) |
| JMP improved latrine | 1579 | 1.05 (0.81,1.37) | 1.44 (0.96,2.17) | 0.96 (0.80,1.16) |

PR = prevalence ratio

^a^ ICDDR,B developed a definition of “hygienic” latrines which differs from the WHO Joint Monitoring Programme (JMP) but may be a more accurate categorization of latrines that isolate feces from the environment for the types of sanitation found in Bangladesh. Hygienic latrines include flush latrines connected to piped sewer system, to septic tank, or off-set pit latrine, pit latrine with slab and functional water seal, pit latrine with slab, lid and no water seal, or a composting latrine. Unhygienic latrines are those that fail to effectively separate feces from the environment: flush latrine connected to canal or ditch, pit latrine without slab, pit latrine with slab, no or broken water seal or a hanging latrine.

This definition differs from the JMP definition in two ways. Hygienic latrines require a water seal or a lid on a pit to effectively separate collected feces from the environment and does not consider sharing status of a latrine. “No access to a latrine” included households who reported no facilities, defecating in open spaces, fields or near water bodies. Field workers also recorded self reports of the latrine ownership and sharing status from the respondents.

^b^ Prevalence ratios were estimated using Poisson regression and adjusted for age, sex, household wealth, cluster-level wealth, geographic district, and mother’s education level.
