# Supplementary material for: The Interaction of Deworming, Improved Sanitation, and Household Flooring with Soil-Transmitted Helminth Infection in Rural Bangladesh
Source: PLoS Negl Trop Dis. 2015 Dec 1;9(12):e0004256. doi: 10.1371/journal.pntd.0004256 (PMC4666415; doi:10.1371/journal.pntd.0004256)
Supplement: S2 Table — (DOCX) [file pntd.0004256.s005.docx]

**S2 Table. Means of each variable used in the principal components analysis by quintile of the index**

|  | Quintile of Wealth | | | | |
| --- | --- | --- | --- | --- | --- |
| Variable | 1 | 2 | 3 | 4 | 5 |
| Electricity | 0.040 | 0.076 | 0.115 | 0.170 | 0.200 |
| Cabinet | 0.015 | 0.045 | 0.112 | 0.176 | 0.198 |
| Table | 0.033 | 0.122 | 0.163 | 0.190 | 0.198 |
| Chair | 0.044 | 0.134 | 0.175 | 0.190 | 0.201 |
| Clock | 0.008 | 0.031 | 0.063 | 0.091 | 0.168 |
| “Khat” bed (higher quality) | 0.039 | 0.077 | 0.135 | 0.186 | 0.199 |
| “Chouki” bed (lower quality) | 0.141 | 0.144 | 0.130 | 0.130 | 0.137 |
| Radio | 0.001 | 0.001 | 0.004 | 0.008 | 0.025 |
| Black and white TV | 0.001 | 0.005 | 0.018 | 0.022 | 0.053 |
| Color TV | 0.000 | 0.007 | 0.015 | 0.062 | 0.137 |
| Refrigerator | 0.000 | 0.000 | 0.002 | 0.014 | 0.057 |
| Bicycle | 0.011 | 0.034 | 0.053 | 0.048 | 0.097 |
| Motorcycle | 0.000 | 0.000 | 0.001 | 0.003 | 0.045 |
| Sewing | 0.004 | 0.005 | 0.016 | 0.018 | 0.056 |
| Mobile phone | 0.083 | 0.138 | 0.170 | 0.194 | 0.201 |
| Sofa | 0.000 | 0.000 | 0.001 | 0.005 | 0.040 |
| Car | 0.000 | 0.001 | 0.002 | 0.004 | 0.010 |
| Land | 0.016 | 0.016 | 0.009 | 0.011 | 0.005 |
| Homestead | 0.178 | 0.189 | 0.190 | 0.198 | 0.199 |
