# Supplementary material for: The Interaction of Deworming, Improved Sanitation, and Household Flooring with Soil-Transmitted Helminth Infection in Rural Bangladesh
Source: PLoS Negl Trop Dis. 2015 Dec 1;9(12):e0004256. doi: 10.1371/journal.pntd.0004256 (PMC4666415; doi:10.1371/journal.pntd.0004256)
Supplement: S3 Table — (DOCX) [file pntd.0004256.s006.docx]

**S3 Table. Missing observations for outcome, exposure, and confounder variables**

|  | Number of missing observations |
| --- | --- |
| Any *Ascaris* eggs | 0 |
| Any hookworm eggs | 0 |
| Any *Trichuris* eggs | 0 |
| Deworming consumption | 8 |
| Access to hygienic latrine | 1 |
| Household has finished floor | 0 |
| Mother's education level | 2 |
| Household wealth | 0 |
| Cluster-level wealth | 0 |
| Geographic district | 0 |
| Age (years) | 15 |
| Sex | 0 |
