# Supplementary material for: The Interaction of Deworming, Improved Sanitation, and Household Flooring with Soil-Transmitted Helminth Infection in Rural Bangladesh
Source: PLoS Negl Trop Dis. 2015 Dec 1;9(12):e0004256. doi: 10.1371/journal.pntd.0004256 (PMC4666415; doi:10.1371/journal.pntd.0004256)
Supplement: S4 Table — (DOCX) [file pntd.0004256.s007.docx]

**S4 Table. Association between SHEWA-B participation and STH infection**

| Helminth | aPR (95% CI) ^a^ |
| --- | --- |
| *Ascaris* | 0.88 (0.69, 1.12) |
| Hookworm | 0.96 (0.64, 1.43) |
| *Trichuris* | 0.94 (0.79, 1.13) |

aPR = adjusted prevalence ratio

^a^ Prevalence ratios were estimated using Poisson regression and adjusted for age, sex, household wealth, cluster-level wealth, geographic division, mother’s education level, deworming in the past 6 months, access to a hygienic latrine, and household floor material
